# Why non-invasive brain stimulation should not be used in military and security services

**DOI:** 10.3389/fnhum.2013.00553

**Published:** 2013-09-09

**Authors:** Bernhard Sehm, Patrick Ragert

**Affiliations:** Department of Neurology, Max Planck Institute for Human Cognitive and Brain Sciences LeipzigLeipzig, Germany

**Keywords:** non-invasive brain stimulation, tDCS, TMS, ethics, military, security

In a recent review article by Levasseur-Moreau et al. (2013), the authors discussed the effects of non-invasive brain stimulation (NIBS) on cognitive functions and proposed a potential application of NIBS in security or military personnel. We believe that this research endeavor is questionable since it might disclose several scientific as well as ethical concerns. In the following, we highlight our reservations about the potential use of NIBS in army and/or security services.

Over the past decades, non-invasive brain stimulation (NIBS) techniques such as transcranial magnetic (TMS) or transcranial direct current stimulation (tDCS) have been extensively used to investigate brain function and brain plasticity in the living human brain. Early studies provided evidence that NIBS is capable of evoking short-lasting modulatory effects on brain functions. Based on this finding, subsequent proof-of-principle studies quickly progressed to also affect motor and cognitive functions. Originally, NIBS techniques were primarily used in basic research to unravel physiological brain processes and/or to establish brain-behavior relationships. The underlying motivation for many researchers is to extend the boundaries of knowledge and to translate findings of basic research into clinical science, that is, to develop new adjuvant therapeutic tools.

In fact, NIBS might be a promising tool for the treatment of neurological and psychiatric diseases (Floel, 2013). For example, Hummel and colleagues showed a beneficial effect of a short period of tDCS in chronic stroke patients on paretic hand function (Hummel et al., 2005). Based on this finding, the authors and other subsequent studies (Lindenberg et al., 2010) suggested that such interventional strategies in combination with customary rehabilitative treatments may play an adjuvant role in neurorehabilitation. Nevertheless, clinical trials on larger patient samples are still needed to confirm the promising results that have been achieved so far by smaller clinical studies.

Apart from a translation to the clinical settings, it has been suggested to use these techniques for “neuroenhancement” in cognitive abilities or sports, fueling a vivid discussion concerning ethical issues of the use of NIBS in healthy human subjects (Hamilton et al., 2011; Brukamp and Gross, 2012; Cohen Kadosh et al., 2012; Banissy and Muggleton, 2013). However, to our mind, the use of these techniques in military or security personnel goes even a step further and accentuates concerns as compared to the use in “civilians.” First, the use of NIBS in military or security services is problematic with respect to the autonomy of individuals receiving NIBS: In the military context, the risk of coercion is much more pronounced and autonomous decisions cannot always be warranted (Tennison and Moreno, 2012). Second, safety issues might be aggravated in this context and might not only apply to the person receiving NIBS but also to third persons. Both safety and autonomy represent principles that may help to identify ethical problems and guide related decisions (Beauchamp and Childress, 1994; Walker, 2009; Brukamp and Gross, 2012).

## What are the long-term effects or side effects of NIBS?

The long-term behavioral effects of NIBS are yet unknown. Single NIBS applications typically result in transient effects on behavior and brain physiology. A few studies, however, indicated, that repeated NIBS applications over several consecutive days during motor or cognitive learning might induce longer-lasting behavioral improvements (Levasseur-Moreau et al., 2013). These findings are certainly of great interest for the application of NIBS in neurorehabilitation, where long-lasting brain changes and associated functional improvements are a desired goal of any treatment. However, we still do not know how specific such changes are and whether improvements in one function may be associated with deterioration in others, as raised by a recent article (Brem et al., 2013). In a clinical setting, patients are under close medical supervision and individually elected for specific treatments, based on a careful assessment of individual risks and benefits. In addition, due to a longitudinal medical monitoring, potential long-term changes may possibly be identified. This, however, does not hold true in military/security context. Therefore, to our mind, it raises ethical questions whether the induction of long-lasting brain changes in healthy individuals, and in particular in military and/or security personnel, should be an aim or even just a tolerated “side effect” of neuroscientific research. Even though hypothetical, the question that comes up is: Do we want to take the risk of changing the brain processing in people who (i) potentially cannot make autonomous decisions concerning the application of NIBS and (ii) are responsible for their own lives as well as the lives of others?

Medical side effects of NIBS described so far in the literature are seldom and usually not severe (with the exception that specific NIBS protocols increase the risk of epileptic seizures). In analoy to unknown long-term effects discussed above, the risk–benefit ratio of NIBS should be carefully evaluated since potential medical risks especially related to repeated brain stimulation are still not well-known (e.g., do repeated applicatons of NIBS increase the risk of epileptic seizures?). Therefore, it certainly cannot be excluded that a repeated exposure to NIBS might result in unforeseeable health issues for the “treated” individual. While this concern is not specific to the application of NIBS in military settings, it again might be especially severe, since the individual might not be able to weigh the risks and benefits and make an autonomous decision (Brukamp and Gross, 2012). On this notion Tennison and Moreno (2012) state that “if a warfighter is allowed no autonomous freedom to accept or decline an enhancement intervention […] then the ethical implications are immense.”

## are the effects of NIBS transferable to the “real world”?

What do we know about the generalization of NIBS-induced effects on everyday life situations? Until now, scientific evidence for NIBS effects has been limited to relatively simplified experimental settings which might not necessarily be valid outside controlled laboratory settings. In order to argue that stimulation of specific brain areas is related to a “meaningful” behavioral effect, researchers usually try to isolate a cognitive process of interest (the dependent variable, e.g., spatial attention) while minimizing or controlling for potential “confounding variables” such as mood changes and so forth. However, there is still limited evidence that NIBS effects can at all be beneficial in real-life situations—where we are subject to complex perceptual, cognitive, and emotional interactions.

Some recent studies investigated the effects of tDCS on visual detection abilities in a task that is specifically designed for military training programs to “familiarize military personnel with the Middle Eastern environment before deployment” (Clark et al., 2012; “*DARWARS Ambush! Threat Detection Task*”). Here, in a so-called “threat detection task” concealed bombs and “enemy combatants” have to be detected in a virtual reality setting that simulates a Middle Eastern environment. While this might be somewhat more realistic, it still remains a computer simulation and surely cannot mimic real-life situations of soldiers and/or security personnel who may need to make fast decisions under extreme and life-threatening conditions with potentially enormous attentional and/or emotional load. We do not know how NIBS techniques affect human behavior in such complex real-life situations. For example, an unwanted and unexpected modulation of the attentional state, decision-making or emotional factors might negatively affect behavioral outcome. Therefore, the use of these techniques must be considered unsafe in particular for third persons that might be harmed by the actions of “dysregulated” individuals receiving NIBS.

## how specific is the modulation of brain function using NIBS?

Despite recent progress, it still remains elusive how specific NIBS protocols act on behavior and/or neural processing. Focal brain stimulation might potentially be suitable for enhancing some abilities in a laboratory setting, but we do not know yet at which costs. As mentioned above, it has been proposed that NIBS performed to enhance a specific ability of interest may be deleterious to another (Hilgetag et al., 2001; Hamilton et al., 2011; Brem et al., 2013). Obviously, due to the limited spatial accuracy of NIBS we do not modulate one segregated brain area that is responsible for one specific function. Instead, recent studies combining NIBS and neuroimaging demonstrate that whole brain functional networks are affected by “focal” stimulation, and increases in functional activity or connectivity of certain brain regions are often accompanied by a decrease in others (Bestmann et al., 2004; Polania et al., 2011; Sehm et al., 2012).

A recent study investigated the effects of tDCS applied over the frontal cortex during a 40-minute vigilance task that was designed to simulate the work of an air traffic controller (Nelson et al., 2012). TDCS over the prefrontal cortex caused a sustained target detection performance thus counteracting a physiological decrease of vigilance in the volunteering military personnel. However, in the same study, tDCS did not only modulate perceptual sensitivity—in the framework of signal detection theory (McMillan, 2005)—but also induced a liberalization in the decision criterion, that is, the internal criterion to differentiate signal from noise. In a similar way, a study by Pavlidou et al. (2012) reported improved visual discrimination of human and animal motion induced by tDCS over premotor cortex but at the costs of an increase in the false alarm rate. However, another study did find a specific effect of tDCS on perceptual sensitivity and no effect on the decision criterion (Falcone et al., 2012). Thus, the results across studies are inconsistent which might depend on differences in NIBS parameters and/or task design. Nevertheless, they question whether only “basic” perceptual abilities are modulated by NIBS or whether additionally the perceptual decision criterion is affected by brain stimulation. This, however, might be an essential issue in military settings. For example, a liberalization of the decision criterion may result in more “hits” but at the costs of more “false alarms.” In the military setting, a “false alarm” that causes a military reaction might have disastrous consequences.

In this context it might be important to consider questions related to the responsibility of individuals undergoing NIBS whose actions harmed themselves or others. Is a soldier that is receiving NIBS responsible for erroneous decisions? Can “wrong” brain stimulation parameters be blamed? These questions still remain unanswered but have tremendous moral and legal implications.

## Conclusion

We here critically discussed the potential application of NIBS in military or security services as proposed in a recent article (Levasseur-Moreau et al., 2013). In our opinion, relevant ethical and scientific concerns as outlined in this article question such implications. In this light, we hope that our arguments will contribute to and stimulate a constructive discussion about the potential use of NIBS in military and/or security services.
